# Invasive non-typhoidal *Salmonella* from stool samples of healthy human carriers are genetically similar to blood culture isolates: a report from the Democratic Republic of the Congo

**DOI:** 10.3389/fmicb.2023.1282894

**Published:** 2023-11-24

**Authors:** Lisette Mbuyi-Kalonji, Liselotte Hardy, Jules Mbuyamba, Marie-France Phoba, Gaëlle Nkoji, Wesley Mattheus, Justin Im, Florian Marks, Hyon Jin Jeon, Jan Jacobs, Octavie Lunguya

**Affiliations:** ^1^Department of Microbiology, National Institute for Biomedical Research, Kinshasa, Democratic Republic of Congo; ^2^Department of Microbiology, University Teaching Hospital of Kinshasa, Kinshasa, Democratic Republic of Congo; ^3^Department of Clinical Sciences, Institute of Tropical Medicine, Antwerp, Belgium; ^4^Department of Microbiology, Immunology and Transplantation, KU Leuven, Leuven, Belgium; ^5^Department of Human Bacterial Diseases, Sciensano, Brussels, Belgium; ^6^International Vaccine Institute, Seoul, Republic of Korea; ^7^Cambridge Institute of Therapeutic Immunology and Infectious Disease, University of Cambridge School of Clinical Medicine, Cambridge Biomedical Campus, Cambridge, United Kingdom; ^8^Heidelberg Institute of Global Health, University of Heidelberg, Heidelberg, Germany; ^9^Madagascar Institute for Vaccine Research, University of Antananarivo, Antananarivo, Madagascar

**Keywords:** *Salmonella Typhimurium* ST313, *Salmonella Enteritidis* ST11, human carriers, reservoir, Africa, DR Congo

## Abstract

Invasive non-typhoidal *Salmonella* (iNTS) (serotypes Typhimurium and Enteritidis) are major causes of bloodstream infections in sub-Saharan Africa, but their reservoir is unknown. Aiming to demonstrate human carriers as a reservoir, we assessed an iNTS disease endemic rural community (Kikonka health area, Democratic Republic of the Congo) for intestinal carriage of iNTS. After a census, healthy subjects from randomly selected households provided three successive stool samples for *Salmonella* culture. We next compared the stool isolates for genetic relatedness with time and health area-matched blood culture isolates obtained from hospitalized patients by multiple locus variable-number tandem repeat analysis (MLVA) and performed whole genome sequencing (WGS) on a subset of stool and blood isolates. Among 2,354 eligible subjects, 2,234 (94.9%) consented and provided at least one stool sample, and 2,219 (94.3%) provided three stool samples. The cumulative proportion of *Salmonella* carriers after 3 days was 4.4% (n = 98). *S*. Typhimurium and Enteritidis were found in 26 and 3 carriers, respectively, representing 1.3% (29 out of 2,234) of participants living in 6.0% (26 out of 482) of households. MLVA types of all 26 *S*. Typhimurium stool isolates matched with the corresponding MLVA types of blood isolates. The MLVA type of one out of three Enteritidis stool isolates matched the single MLVA type of the five Enteritidis blood isolates. WGS analysis of *S*. Typhimurium (n = 20) and *S*. Enteritidis (n = 4) isolates revealed Typhimurium multilocus sequence type (ST)313 Lineage 2 and Enteritidis ST11 Central/Eastern African and Outlier clades and confirmed the MLVA clustering. More than three-quarters of Typhimurium isolates showed combined multidrug resistance, ceftriaxone resistance, and fluoroquinolone non-susceptibility. In conclusion, the present study demonstrated iNTS carriage among healthy community members, with stool isolates that were genetically similar to blood culture isolates obtained in patients from the same community. These findings contribute to the evidence of a human reservoir of iNTS.

## Introduction

Non-typhoidal *Salmonella* are a major cause of invasive infections in sub-Saharan Africa, particularly among children younger than 5 years old with *Plasmodium falciparum* malaria or malnutrition and HIV-infected adults (Feasey et al., 2012; Crump and Heyderman, 2015; Gilchrist and MacLennan, 2019). For the year 2017, the burden of invasive non-typhoidal *Salmonella* (iNTS) infections in sub-Saharan Africa was estimated to be 421,600 cases (95% confidence interval (C.I.) 316,000 to 574,100) resulting in 66,520 deaths (40,130 to 105,500) (Stanaway et al., 2019). The most frequent iNTS serotypes are *Salmonella enterica* subspecies *enterica* serotype Typhimurium and Enteritidis, respectively (further shortly referred to as *S*. Typhimurium and *S*. Enteritidis). Typical clades in sub-Saharan Africa are *S*. Typhimurium multilocus sequence type (ST) 313 (in particular the multidrug-resistant Lineage 2) and the Central/East-African and West-African subclades of *S*. Enteritidis ST11 (Feasey et al., 2016; Van Puyvelde et al., 2019; Park et al., 2021; Pulford et al., 2021).

*Salmonella* Typhi and Paratyphi (causing enteric fever) are human-specific, and human intestinal carriers (*i.e.*, convalescent or healthy subjects shedding *S*. Typhi/Paratyphi in their stools) have been identified as the reservoir; transmission occurs through contaminated water or food (Crump and Heyderman, 2015). Conversely, the reservoir and transmission of iNTS are not fully understood. Given their broad host range, *S*. Typhimurium and *S*. Enteritidis are categorized as “generalist” serovars and their reservoir is assumed to be zoonotic (Feasey et al., 2012; Crump and Heyderman, 2015), but invasive *S*. Typhimurium and *S*. Enteritidis underwent genome degradations such as pseudogenes and deletions, which could be consistent with adaptation to human host (Feasey et al., 2016; Pulford et al., 2021) and consistent arguments for a human reservoir have been provided (Kariukiet al., 2006; Dione, 2010; Post et al., 2019; Phoba et al., 2020).

In the Democratic Republic of the Congo (DRC), the National Institute of Biomedical Research (INRB, Kinshasa) in partnership with the Institute of Tropical Medicine (ITM, Belgium) has a microbiological surveillance network in place since 2007. Laboratories of sentinel hospitals across DRC process free-of-charge blood cultures implemented in routine patient care. Kisantu General Referral Hospital Saint Luc (further referred to as Kisantu Hospital) in the Province of Kongo Central is the main sampling site. Over the years 2015–2017, iNTS accounted for >70% of blood culture pathogens recovered in children younger than 5 years old admitted to Kisantu Hospital, representing nearly 300 patients per year (Tack et al., 2020a). From October 2017 to March 2020, Kisantu Hospital was the DRC study site of the Severe Typhoid in Africa (SETA) program coordinated by the International Vaccine Institute (IVI, Republic of Korea). The SETA program conducted enhanced surveillance for febrile illnesses in sites of six sub-Saharan African countries (Hyon Jin Jeon submitted).

Recently, we assessed non-typhoidal *Salmonella* intestinal carriage in healthy subjects living in a *Schistosoma*-endemic area in the Kongo Central Province of DRC, at a distance of 66 km from Kisantu. Among 38 *Salmonella* isolates (overall prevalence of 3.4%), there were 4 and 5 Typhimurium and Enteritidis isolates, respectively (Mbuyi-Kalonji et al., 2020). Most of these isolates were genetically similar to Typhimurium and Enteritidis isolates obtained from blood cultures in Kisantu Hospital. Furthermore, previous studies (Burkina Faso, DRC) showed among non-typhoidal *Salmonella* a tendency toward household clustering (Im et al., 2016; Post et al., 2019; Falay et al., 2023). We hypothesized that sampling a larger community closer to Kisantu Hospital would allow for a better study of the concordance between blood and stool culture iNTS isolates and their occurrence in households. We therefore conducted a household-level randomized stool culture in a community close to Kisantu Hospital. The objectives were to assess (i) the proportion, age, and gender distribution of human *Salmonella* intestinal carriers (in particular iNTS); (ii) the serotype distribution and antimicrobial resistance (AMR) pattern of the isolates; and (iii) the genetic relatedness between iNTS stool and blood culture isolates obtained from Kisantu Hospital. Secondary objectives were to assess the incremental yield of three consecutive stool samples for the detection of NTS carriers and the potential clustering of *Salmonella* carriers among household members.

## Methods

### Ethics statement

This study was approved by the Institutional Review Board of the Institute of Tropical Medicine (Reference 1263/18), the Ethical Committee of the University of Antwerp (Reference 18/47/535), and the Ethical Committee of Public Health School in Kinshasa, Democratic Republic of the Congo (ESP/EC/070/2019). Adults were included after written informed consent was provided. All children younger than 18 years old were included after written informed consent from their parents or their legal guardians. Additionally, for adolescents between 12 and 18 years of age, a written assent was obtained. Ethical approval for the blood culture surveillance study was granted by the Institutional Review Board of the Institute of Tropical Medicine (Reference 08 17 12 613) and the Ethics Committee of Public Health School in Kinshasa (version 1: ESP/CE/073/2015 and version 2: ESP/CE/074/2015). For the SETA study, ethical approvals were granted by the International Vaccine Institute Institutional Review Board (IRB No. 2015-006) and the Ethics Committee of Public Health School in Kinshasa (version 1.1: ESP/CE/011/2017, version 1.2: ESP/CE/011B/2017, and version 1.3: ESP/CE/037/2018 and ESP/CE/037B/2019). Compensation for participation in the study was provided to the participating community, and it was used to procure a plot of land on which a new and large health center for Kikonka area will be built.

### Study design

The study design consisted of a cross-sectional community-based study, with a census-based randomized selection of households of which healthy members (participants) were assessed for *Salmonella* intestinal carriage by microbiological culture of three consecutive stool samples. *Salmonella* Typhimurium and Enteritidis stool isolates were compared for genetic relatedness with blood culture isolates obtained from patients of the same community who were hospitalized during the study period.

### Study site

The study was conducted in the Province of Kongo Central (western DRC), in Kikonka health area (further referred to as “Kikonka”), located at a distance of 7 km from Kisantu Hospital. Details about the health and population indicators of Kisantu health zone are presented in reference (Tack et al., 2020a). A census was conducted for the preparation of this study.

### Households, study participants, and study period

Households were defined as subjects (mostly family members), sharing the same kitchen and sanitation and recognizing the authority of a household head. All residents of Kikonka who were >29 days old had provided consent, were healthy, and were eligible for participation. Healthy status was defined as the absence of a recent history of fever (≤ 14 days), diarrhea (24 h before enrollment), and antibiotic treatment (≤ 48 h before enrollment) (Mbuyi-Kalonji et al., 2020). A participant was defined as an eligible household member after consenting to the participation in the study and providing at least one stool sample. The study period extended from September 2019 to March 2020 during the rainy season.

### Sample size and selection of households

Based on our previous study in Kongo-Central DRC, a minimum of 60 non-typhoidal *Salmonella* stool culture isolates were targeted to obtain reliable data on serotype distribution, AMR profiles, and genetic relatedness with blood culture isolates. Given the prevalence of *Salmonella* carriage (3%) observed during that study (Mbuyi-Kalonji et al., 2020), the target sample size was calculated to be 2,000 participants. Estimating that 20% (*n* = 400) of participants would not submit second and third stool samples, the sample size was increased to 2,400 participants. Based on an anticipated household size of four to five members per household, 450 households were foreseen. Each seventh household in the list of all households of Kikonka (*n* = 3,128, counted by the census) was selected. Selected households were representative of the geographical distribution of the population.

### Recruitment, sample collection, and interviews

After the approval of the study by the local health and community authorities, sensitization of the Kikonka population was performed through local television and radio stations and door-to-door visits by community health workers. For recruitment, study teams visited the selected households early in the morning. After consenting, participants were asked to provide three consecutive stool samples within 1 week. Age, gender, and household location by GPS coordinates were recorded, and participants (or their caretakers) were interviewed about demographics and health status. After the first round of recruitment, a second round was organized to catch up with the sample size for subjects who were not available at the first visit or who had consented but not yet provided a stool sample.

### Stool culture for *Salmonella*

The day after the study visit, stool samples were collected by community health workers. Within 2–8 h after production, the samples were transported to the Kikonka Health Center using cool boxes (2–8°C). At Kikonka Health Center, ~1 g of each stool sample was inoculated into 10 ml of Selenite broth (BD Difco, Becton Dickinson and Company, NJ, US). Selenite broth samples were transported to the laboratory of the Kisantu Hospital where they were incubated at 36°C. After 18 to 24 h of incubation, 10 μl of Selenite was subcultured on two plates of CHROMagar^TM^ *Salmonella* (CHROMagar^TM^, Paris, France, a selective medium for color-based detection of *Salmonella*). After incubation (18–24 h at 36°C), purple colonies (*i.e.*, indicative of *Salmonella*) were subcultured on Kligler Iron Agar (KIA) (Oxoid Ltd., Basingstoke, Hampshire, England) with a maximum of four colonies per plate. If present, different colony types were subcultured (two colonies per type). When no purple colonies were observed, the plates were incubated for another 24 h and read after 48 h of incubation.

### Identification, serotyping, and antibiotic susceptibility testing

Isolates indicative of *Salmonella* were tested biochemically (DiaTabs, Rosco, Taastrup, Denmark) (Mbuyi-Kalonji et al., 2020) and, if confirmed, stored in tubes with Tryptone Soya Agar (TSA, Oxoid) and shipped to INRB and ITM for serotyping (Pro-lab Diagnostics Inc., Richmond Hill, Ontario, Canada) and antibiotic susceptibility testing. Antibiotic susceptibility testing was performed by the disk diffusion method (Neo-Sensitabs, Rosco) (CLSI, 2022b), in addition to the assessment of ciprofloxacin and azithromycin minimal inhibitory concentration (MIC) values by the E-test macromethod (bioMérieux, Marcy L'Etoile, France). The CLSI breakpoint of azithromycin susceptibility testing for *Salmonella* Typhi was used for NTS isolates (Tack et al., 2022). Multidrug resistance (MDR) was defined as co-resistance to ampicillin, chloramphenicol, and trimethoprim-sulfamethoxazole. Decreased ciprofloxacin susceptibility (DCS, equivalent to the CLSI intermediate susceptibility category) was defined as ciprofloxacin MIC values of > 0.064 μg/ml and < 1 μg/ml, ciprofloxacin resistance was defined as MIC-values of ≥ 1 μg/ml. Fluoroquinolone non-susceptibility (FQNS) comprises both DCS and ciprofloxacin resistance (Tack et al., 2020b). MIC_50_ (MIC required to inhibit 50% of tested isolates) was used to describe differences between ciprofloxacin MIC values of different serotypes (CLSI, 2022a).

### Comparison between stool culture isolates and blood culture isolates

Blood culture iNTS isolates obtained between September 2019 and March 2020 from patients living in Kikonka were retrieved [for indications and laboratory work-up, see reference (Tack et al., 2020a)]. Comparison with stool cultures was carried out by multiple locus variable-number of tandem repeat analysis (MLVA) as described previously (Mbuyi-Kalonji et al., 2020). For *S*. Typhimurium, MLVA clusters were defined as isolates with none or one variation in one of the non-stable loci (STTR5, STTR6, and STTR10) and no variation in the stable loci (STTR3 and STTR9) (Dimovski et al., 2014). For *S*. Enteritidis, MLVA clusters were defined as isolates with none or one variation in one of the five loci (Hopkins al., 2011).

For assessing ST lineages, a subset of isolates was selected according to representativity (stool vs. blood culture isolates) and the different MLVA clusters. WGS was performed by the commercial genomic platform of Eurofins Genomics (Konstanz, Germany). DNA extraction and purification from bacterial isolates were performed by Eurofins, as well as library preparation and sequencing (Illumina, San Diego, CA, USA), generating 150 bp paired-end reads. The short reads were assembled *de novo* with SPAdes version 3.6.0.23. Various tools integrated into EnteroBase (REF in footnote)^1^ were used for sequence analysis, as described previously (Zhou et al., 2021; Falay et al., 2022). A minimum spanning (MS) tree (MStree V2 using GrapeTree) based on the EnteroBase 3002 loci “cgMLST V2 + HierCC V1” scheme was produced for each serotype to estimate the allelic distances between isolates from this study and the genomes from references (Feasey et al., 2016; Pulford et al., 2021).

### Definitions, data collection, and analysis

The definition of participants is mentioned above. *Salmonella* carriers were defined as participants with growth of *Salmonella* from at least one stool sample. Household *Salmonella* clusters were defined as ≥ 2 carriers living in the same household, for whom the same *Salmonella* serotype was isolated from at least one stool sample; in the case of *S*. Typhimurium and Enteritidis, an identical or similar MLVA profile was added as a criterium.

Data from paper-based interviews and laboratory data were encoded in and analyzed by Excel 2301 (Microsoft, Richmond, VA, US). Serotyping and MLVA typing were performed on all subsequent *Salmonella* isolates per participant. Data (assessed in Excel) were described by ratios with 95% C.I. and medians with ranges. Differences between proportions were assessed for statistical significance (defined as *p*-value < 0.05) using chi-square or Fisher exact tests.

## Results

### Study population and representativeness participation rates

**Figure 1:**
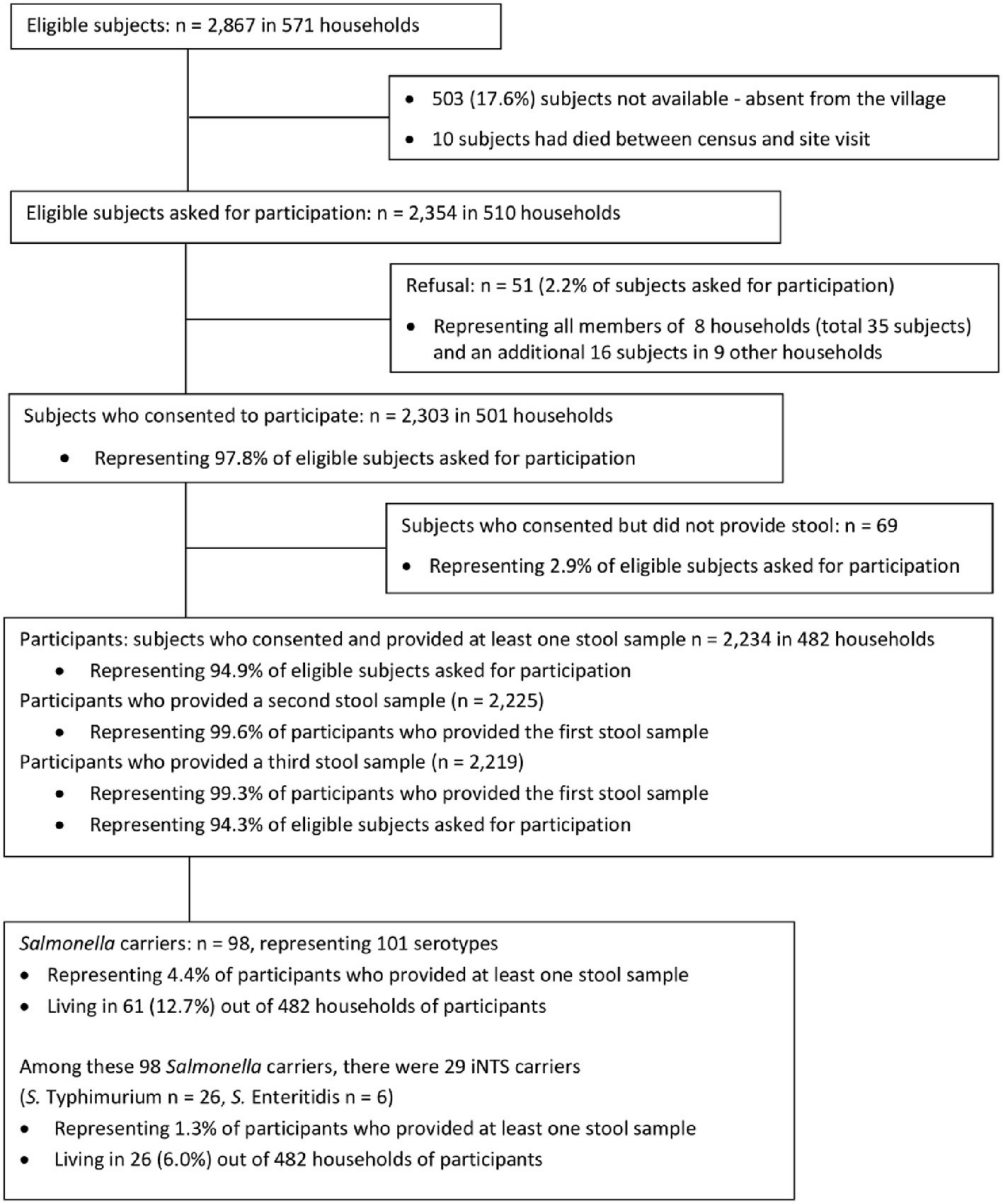
Breakdown of the study population (Testing Rounds 1 and 2 combined).

The census revealed 16,503 inhabitants living in 3,218 households, with a population density of 82.5 inhabitants/km^2^. A total of 571 households comprising 2,867 eligible subjects were visited, of whom, 513 were not available (Figure 1). Among 2,354 eligible subjects asked for participation, 2.2% refused and another 2.9% consented but did not provide a stool sample, resulting in 94.9% (*n* = 2,234) participants who provided at least one stool sample. Their median age was 16 years (30 days−91 years); the male-to-female ratio was 1:1.08. Households comprised a median of 5 (1 – 12) members. These data were representative of the total Kikonka population and similar to those of eligible subjects who were not available during the study visits (Supplementary Tables 1–3). Nearly all participants (99.3%, 2219 out of 2234) provided three samples; 93.4% (2072/2219) of participants submitted three stool samples within 1 week. Two (0.09%) participants reported the use of antibiotics within 48 h prior to inclusion, and one (0.04%) participant reported a history of diarrhea.

### Proportions and demographics of *Salmonella* carriers

A total of 98 out of 2,234 (4.4% 95% CI: 3.6–5.4%) *Salmonella* intestinal carriers were detected, living in 61 out of 482 (12.6%) households (Figure 1). Their median age was 13 years (4 months−75 years) with a male-to-female ratio of 1:0.92. The proportion of *Salmonella* carriers among children of < 15 years old (5.0%, 52/1,033; 95% CI: 3.9–6.5) was higher compared with adults (3.8%, 46/1,201; 95% CI: 2.9–5.1), but this difference did not reach statistical significance (*p* = 0.166) (Table 1). *Salmonella* carriers tended to be slightly more frequent among male subjects (51 out of 1,072) (4.8%, 95% CI: 3.6–6.2) than among female subjects (47 out of 1162) (4.0%, 95% CI: 3.0 – 5.3) (*p* = 0.411).

**Table 1:** Distribution of serotypes among *Salmonella* carriers and numbers of isolates per age group.

**Age groups (n)/ *Salmonella* serotypes within the age group**	**Numbers of *Salmonella* carriers**	**Numbers of isolates**	**Proportions of *Salmonella* carriers within the age groups**
Children < 5 years (*n* = 355)	20	21	5.6%
*Salmonella* Enteritidis		2	
*Salmonella* Kentucky		6	
*Salmonella* Typhimurium		9	
*Salmonella* Urbana		2	
*Salmonella* II 42:r:-		2	
Children 5– < 15 years (*n* = 678)	32	34	4.7%
*Salmonella* Kentucky		20	
*Salmonella* Typhimurium		9	
*Salmonella* Urbana		2	
*Salmonella* II 42:r:-		3	
Adults ≥ 15 years (*n* = 1,201)	46	46	3.8%
*Salmonella* Enteritidis		1	
*Salmonella* Kentucky		23	
*Salmonella* Typhimurium		8	
*Salmonella* Typhi		1	
*Salmonella* Urbana		1	
*Salmonella* II 42:r:-		10	
*Salmonella* Tempe		1	
*Salmonella* I 11:-:1,2		1	
Total number of participants (*n* = 2234)	98	101	4.4%

### Serotype distribution of *Salmonella* intestinal isolates

The 98 *Salmonella* carriers comprised 101 non-duplicate isolates, as 3 out of 98 carriers had two distinct serotypes. *Salmonella* Kentucky and *S*. Typhimurium were found in half (49 out of 98, 50.0%) and a quarter (26 out of 98, 26.5%) of *Salmonella* carriers (Table 2). Four Typhimurium isolates belonged to the variant Copenhagen (*i.e.*, antigen O5-negative). *Salmonella* II 42:r:- (*Salmonella enterica* subspecies *salamae*) was found in 15.3% (15 out of 98) of *Salmonella* carriers, and the other serotypes included Urbana (*n* = 5 carriers), Enteritidis (*n* = 3), Tempe, Typhi, and *Salmonella* I 11:-:1,2 (*n* = 1 each). Apart from *Salmonella* II 42:r:-, for which 10 out of 15 isolates were found among adults, there was no particular association between age group and serotype (Table 1).

**Table 2:** Numbers of serotypes isolated from *Salmonella* carriers.

**Serotypes**	**Numbers of carriers**	**Number of households per serotype**	**Numbers of isolates (% of total)**
*Salmonella* Kentucky	49	25	49 (48.5)
*Salmonella* Typhimurium	26	19	26 (25.7)^*^
*Salmonella* II 42:r:-	14	12	15 (14.9)
*Salmonella* Urbana	3	4	5 (4.9)
*Salmonella* Enteritidis	3	3	3 (2.9)
*Salmonella* Tempe	1	1	1 (0.9)
*Salmonella* Typhi	1	1	1 (0.9)
*Salmonella* I 11:-:1,2	1	1	1 (0.9)
Total	98	61	101 (100.0)

^*^Including four isolates of variant Copenhagen.

### Antibiotic susceptibility profile of *Salmonella* stool culture isolates

All but one (25 out of 26, 96.2%) *S*. Typhimurium intestinal isolates were MDR. Most (80.8%, 21 out of 26) displayed FQNS; their MIC50 (MIC value at which 50% of isolates were inhibited) for ciprofloxacin was 1 (range 0.75–1.5) μg/ml); 3 and 18 of them showed decreased ciprofloxacin susceptibility and ciprofloxacin resistance. Furthermore, they were FQNS, MDR, and resistance to third-generation cephalosporins (Table 3). In total, 1 out of 3 *S*. Enteritidis was MDR; the single *S*. Typhi isolate was resistant to trimethoprim-sulfamethoxazole. Nearly all (48 out of 49, 97.9%) *S*. Kentucky isolates were resistant to trimethoprim-sulfamethoxazole, chloramphenicol, and ciprofloxacin; MIC50 value for ciprofloxacin was 8 (4–24) μg/ml. The remaining *Salmonella* serotype isolates did not display AMR.

**Table 3:** Antibiotic resistance profiles of the non-typhoidal *Salmonella* from stool and blood culture isolates.

	**Stool culture isolates (*****n*** = **101)**				**Blood culture isolates(*****n*** = **57)**	
**Antibiotics**	***Salmonella*** **Kentucky (*****n*** = **49)**	***Salmonella*** **Typhimurium (*****n*** = 26^*^)	***Salmonella*** **Enteritidis (*****n*** = **3)**	**Other serotypes (*****n*** = 23^**^)	***Salmonella*****Typhimurium (*****n*** = 52^***^)	***Salmonella*** **Enteritidis (*****n*** = **5)**
Ampicillin	0	26	1	0	51	5
Trimethoprim-sulfamethoxazole	48	25	1	1^**^	48	5
Chloramphenicol	48	25	1	0	48	5
Ciprofloxacin (DCS/Cip-R)	48	3/18	0	0	3/39	0
Ceftriaxone (C3G-R)	0	21	0	0	44	0
Azithromycin	0	0	0	0	0	0
Meropenem	0	0	0	0	0	0
Gentamicin	0	23	0	0	44	0
MDR	0	25	1	0	47	5
FQNS	48	21	0	0	42	0
MDR + FQNS	0	21	0	0	40	0
MDR + C3G-R	0	21	0	0	42	0
MDR + C3G-R + FQNS	0	21	0	0	40	0

Numbers express the numbers of intermediate-susceptible and resistant isolates combined. Abbreviations and definitions: C3G-R: resistance to third-generation cephalosporins; DCS: decreased ciprofloxacin susceptibility, equivalent to intermediate susceptibility (Minimal Inhibitory Concentration (MIC) values: >0.064 and <1 μg/ml), Cip-R, resistance to ciprofloxacin (MIC values: ≥ 1 μg/ml); FQNS, fluoroquinolone non-susceptibility (comprised both DCS and Cip-R); C3G-R, Resistant to third-generation cephalosporins; MDR, multidrug-resistant, i.e., co-resistant to ampicillin, trimethoprim-sulfamethoxazole, and chloramphenicol.

^*^Including *Salmonella* Typhimurium variant Copenhagen (n = 4).

^**^Including *Salmonella* Typhi (n = 1).

^***^Including *Salmonella* Typhimurium var Copenhagen (n = 5).

### Invasive non-typhoidal *Salmonella* from blood cultures

Blood culture isolates used for comparison included 52 *S*. Typhimurium and 5 *S*. Enteritidis isolates obtained in 56 patients, most of whom (*n* = 54, 96.4%) were younger than 5 years old. *S*. Typhimurium isolates displayed similar proportions of AMR as the stool culture isolates, with 76.9% (40/52) isolates combining MDR, third-generation cephalosporin resistance, and FQNS, the latter mostly ciprofloxacin resistance (Table 3).

### Genetic relatedness between stool and blood culture iNTS isolates and sequence types

*S*. Typhimurium isolates from stool and blood cultures comprised 7 and 16 MLVA types, respectively (Table 4). The MLVA types of all 26 *S*. Typhimurium stool culture isolates matched with the corresponding MLVA types from blood culture isolates. MLVA type 2-9-12-7-0210 represented 15 out of 26 stool isolates and 29 out of 52 blood culture isolates, respectively, and occurred throughout the study period, whereas other MLVA types were more time-bound. The stool and blood culture isolates with time-bound MLVA types 2-5-15-8-0210, 2-NA-12-7-0210, and 2-NA-16-8-0210 were coinciding in time and these isolates belonged to the *S*. Typhimurium variant Copenhagen.

**Table 4:** Multi-locus variable-number tandem repeat analysis (MLVA) types of *Salmonella* Typhimurium isolates from stool cultures (n = 26 carriers, upper panel) vs. isolates from blood cultures (n = 52 hospital admitted patients, lower panel) along the study period.

	**October 2019**	**November 2019**	**December 2019**	**January 2020**	**February 2020**	**March 2020**	**Total**
**MLVA stool culture isolates**							
2-5-15-8-0210		2^*W^	1^*^	0			3
2-9-12-7-0210	1^W^	7 ^W^	1	0	2^W^	4^W^	15
2-9-12-8-0210	1			0			1
2-10-12-7-0210			2^W(2)^	0			2
2-10-13-7-0210		1^W^	2	0			3
2-NA-12-7-0210		1^*W^		0			1
2-NA-16-8-0210			1^*W^	0			1
Total	2	11	7	0	2	4	26
**MLVA blood culture isolates**							
2-4-12-7-0210			1^*^				1
2-5-10-7-0210					1		1
2-5-15-8-0210			1^*W^			1^*^	2
2-8-12-8-0210		1					1
2-9-11-7-0210					1		1
2-9-12-7-0210	7^W^	5^W(2)^	6^W^	5	6^W^	2^W^	31
2-9-12-8-0210		1			1		2
2-9-13-7-0210			1				1
2-9-NA-7-0210			1				1
2-10-12-7-0210	1		1				2
2-10-12-NA-0210				1			1
2-10-13-7-0210				1^W^	3	1	5
2-10-13-8-0210		1					1
2-NA-12-7-0210		1^*W^					1
2-NA-16-8-0210			1^*W^				1
Total	8	9	12	7	12	4	52

The shade of colors indicates clusters of MLVA types.

^*^Salmonella Typhimurium variant Copenhagen.

^W^*Salmonella* isolates selected for whole-genome sequencing analysis (n = 20).

*S*. Enteritidis stool (*n* = 3) and blood culture (*n* = 5) isolates belonged to 3 and 1 MLVA types, respectively. The blood culture MLVA type 2-15-3-3-NA occurred throughout the study period, and one stool culture isolate shared this MLVA type which was MDR. The other three MLVA types from Enteritidis stool cultures were clearly distinct and were susceptible to all antibiotics tested (Table 5).

**Table 5:** Multi-locus variable-number tandem repeat analysis (MLVA) types of *Salmonella* Enteritidis isolates from stool culture (*n* = 3 carriers, upper panel) vs. isolates from blood cultures (*n* = 5 hospital admitted patients, lower panel) along the study period.

	**October 2019**	**November 2019**	**December 2019**	**January 2020**	**February 2020**	**March 2020**	**Total**
**MLVA stool culture isolates**							
2-12-4-6-1	1^W^						1
2-15-3-3-NA		1^W^					1
2-9-7-3-2					1^W^		1
Total	1	1	0	0	1	0	3
**MLVA blood culture isolates**							
2-15-3-3-NA	1^W^			3		1	5
Total	1	0	0	3	0	1	5

^W^Salmonella isolates selected for whole-genome sequencing analysis.

The shade of colors indicates clusters of MLVA types.

**Figure 2:**
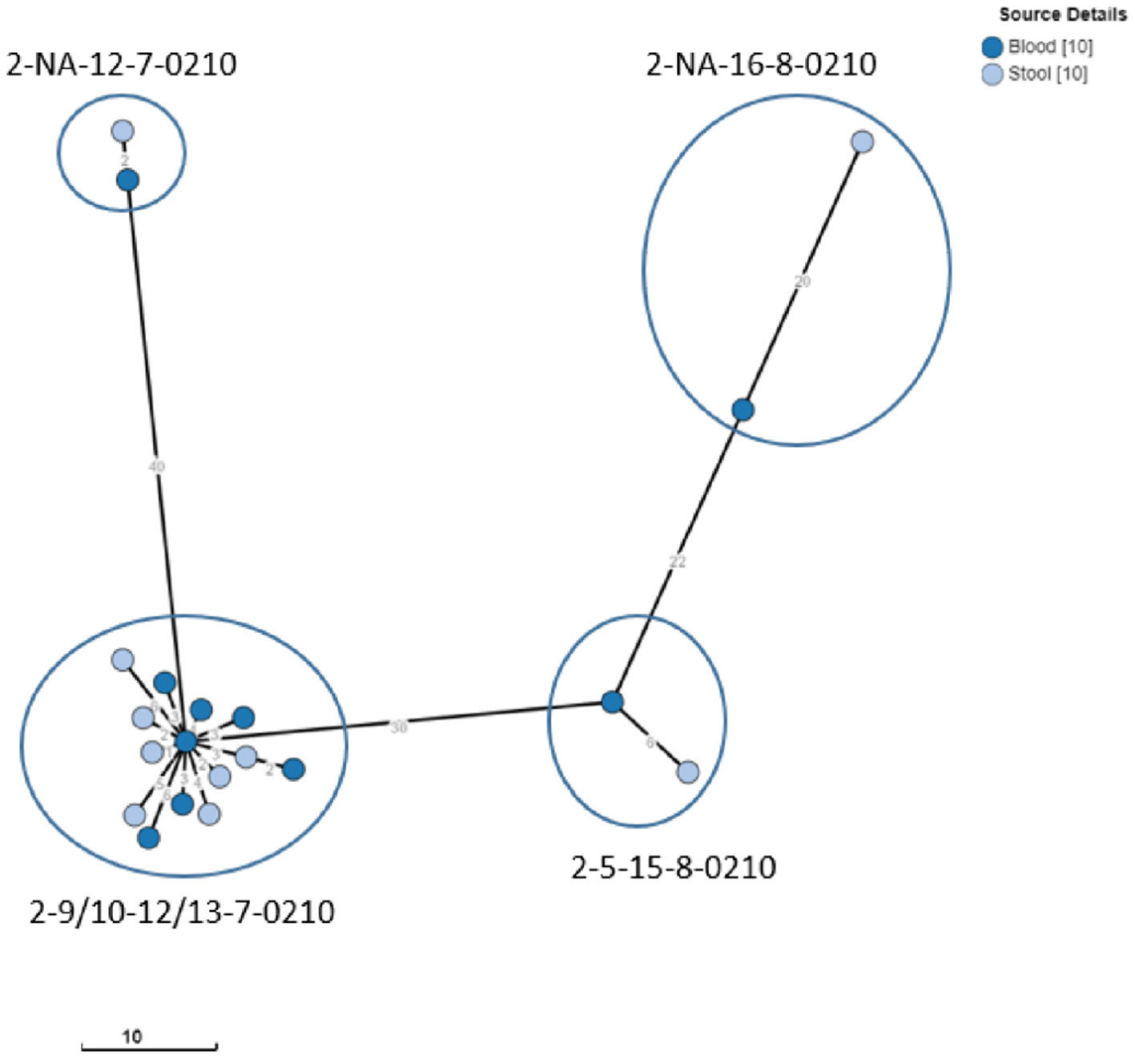
Clustering of S. Typhimurium ST313 from blood and stool cultures. Minimum spanning tree created using the MSTree V2 component in EnteroBase, based on the allelic differences over the 3,002 alleles that constitute the EnteroBase HierCC scheme on cgMLST (Zhou et al., 2021). The distances between leaves in the tree indicate the number of alleles which are different between genomes. Node colors are according to isolate origin. Isolates with the same MLVA type are circled.

The iNTS isolates selected for WGS included 20 *S*. Typhimurium and 4 *S*. Enteritidis isolates obtained from blood and stool cultures (*n* = 11 and 13, respectively), representing all MLVA clusters (Tables 4, 5). WGS revealed that all *S*. Typhimurium isolates belonged to ST313 Lineage 2 as described by Pulford et al. (2021). Isolates with the most frequently observed MLVA type 2-9/10-12/13-7-0210 formed a tight cluster [0–7 allelic differences (AD)] of seven stool culture and seven blood culture isolates. The three other Typhimurium stool culture isolates clustered with the selected blood culture isolates of the same MLVA type (Figure 2). Two of the Enteritidis isolates (one blood culture and one stool) belonged to ST11 of the Central/Eastern African clade (HierBAPS clade 9, HC50_12675) as described by Feasey et al. (2016) and clustered with 13 AD, whereas the two other stool isolates belonged to ST11 of the outlier clade [previously described by Kariuki et al. (2020)] and were non-related (>200 AD).

### Frequency, age, and gender distribution of iNTS carriers

Overall, iNTS carriers represented 29 (1.3%) of participants in 6.0% (26 out of 482) of households. Their median age was 8 years (4 months−64 years), with a male-to-female ratio of 1:0.61. Of all 29 carriers, 11 were younger than 5 years old, 9 were between 5 and 15 years old, and 10 were adults (Table 1).

### Clustering and geographic distribution of *Salmonella* carriers

**Figure 3:**
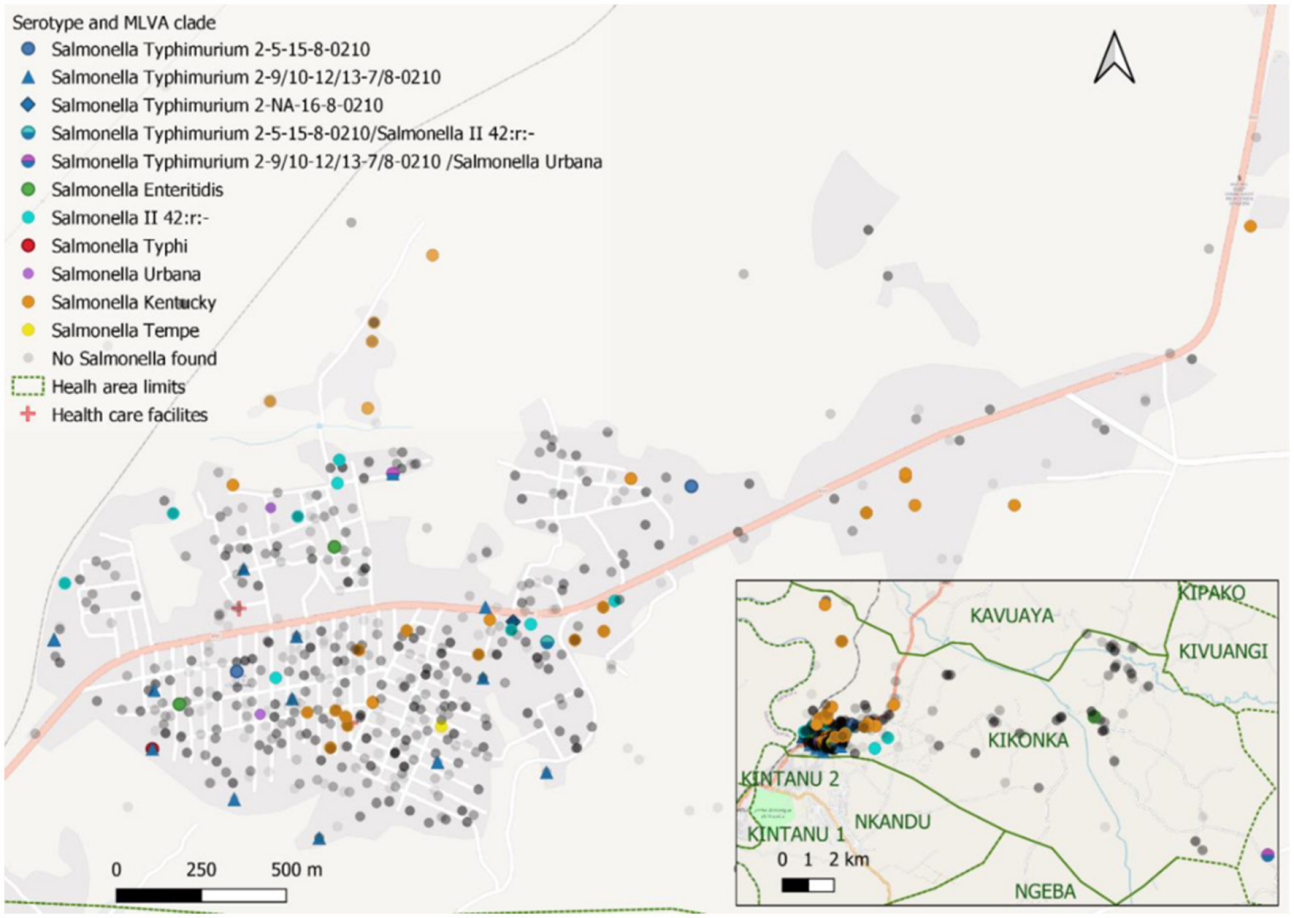
Distribution of study participants and *Salmonella* cases in the Kikonka health area. The pink line represents the main road across the Kikonka health area (National Road N°1, between the Atlantic coast and Kinshasa). Gray dots represent the households that participated in the study, with the intensity of gray corresponding to the number of households at a given place. *Salmonella* serovars are displayed using dots with different colors. Dots containing two colors represent *Salmonella* carriers with multiple isolates/serotypes.

Among the 61 households with *Salmonella* carriers, approximately one-third (*n* = 20, 32.8%) had clusters of two or more carriers of the same serotype, comprising more than half (55 out of 98, 56.1%) of carriers (Table 6). The median number of *Salmonella* carriers per cluster was 2 (2–7); 14 clusters comprised *S*. Kentucky carriers (the largest composed of 5 carriers) and 2 clusters comprised *S*. Typhimurium carriers (the largest included all 7 household members belonging to MLVA type 2-9-12-7-0210). Participating households including those with *Salmonella* carriers were mostly concentrated around the National Road N°1, which canalizes the traffic between the Matadi seaport and the capital, but no particular geographic pattern was observed for any of the serotypes (Figure 3).

**Table 6:** Household clusters (*n* = 20) in which more than one *Salmonella* carrier with an identical serotype was detected for 55 *Salmonella* carriers in the Kikonka health area.

**Households clusters (*n* = 20)**	**Serotypes**	**Number of *Salmonella* carriers (*n* = 55)**	**Date(s) of sampling**
Household KIK0165	*Salmonella* Typhimurium	7	11/11/2019
Household KIK1870	*Salmonella* II 42:r:-	2	02/12/2019
Household KIK2118	*Salmonella* II 42:r:-	2	09/12/2019
Household KIK1036	*Salmonella* Urbana	2	23/12/2019
Household KIK1316	*Salmonella* Kentucky	2	06/01/2020
Household KIK1387	*Salmonella* Kentucky	2	13/01/2020
Household KIK2180	*Salmonella* Kentucky	3	13/01/2020
Household KIK2487	*Salmonella* Kentucky	4	13/01/2020
Household KIK2493	*Salmonella* Kentucky	5	13/01/2020
Household KIK1335	*Salmonella* Kentucky	2	13 - 15/01/2020
Household KIK2499	*Salmonella* Kentucky	3	13 - 16/01/2020
Household KIK2557	*Salmonella* Kentucky	2	20/01/2020
Household KIK2657	*Salmonella* Kentucky	3	20/01/2020
Household KIK2683	*Salmonella* Kentucky	2	20/01/2020
Household KIK2811	*Salmonella* Kentucky	2	20/01/2020
Household KIK2543	*Salmonella* Kentucky	2	20 - 21/01/2020
Household KIK2663	*Salmonella* Kentucky	3	20 - 21/01/2020
Household KIK2671	*Salmonella* Kentucky	3	21/01/2020
Household KIK2626	*Salmonella* II 42:r:-	2	27/01/2020
Household KIK0322	*Salmonella* Typhimurium	2	16 - 17/03/2020

The first column refers to the code number of the household. Clusters are chronologically ranked.

### Incremental yield of three consecutive stool samples and use of the CHROMagar^*TM*^ *Salmonella* medium

**Figure 4:**
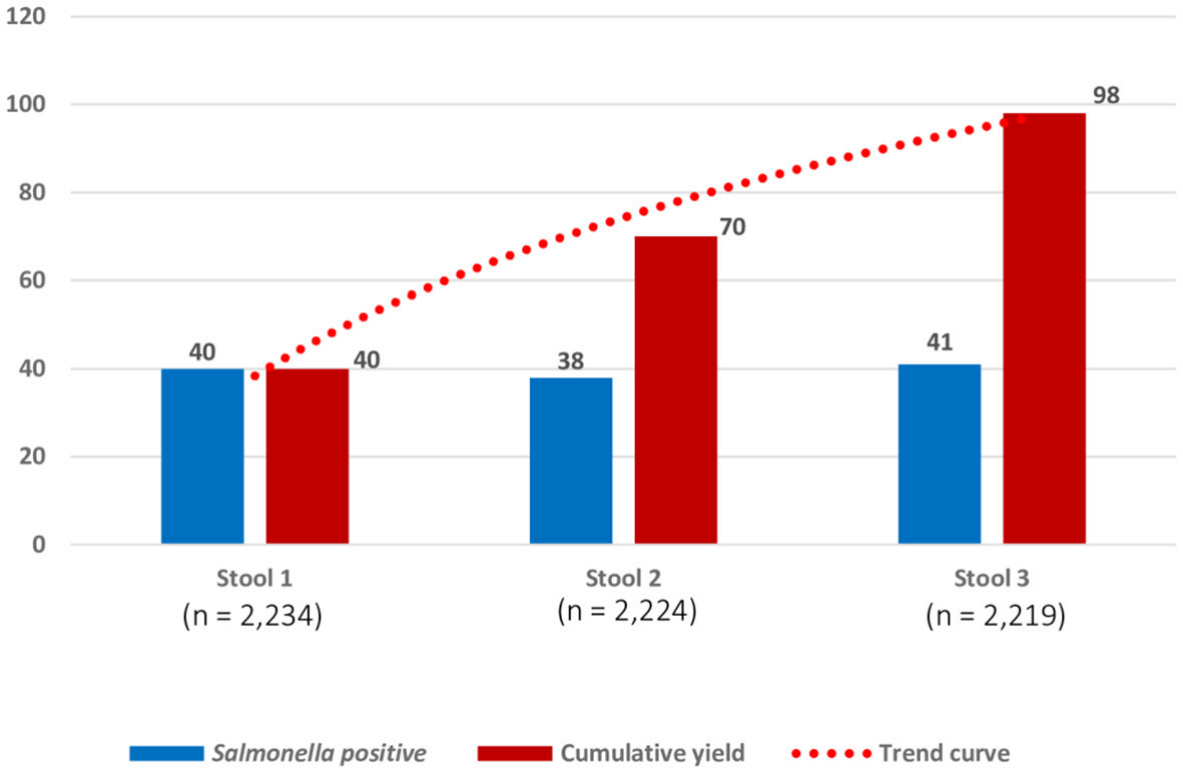
Numbers of successive stool samples grown with Salmonella for 2,219 participants who provided three consecutive stool samples. Numbers (Y-axis) represent samples with the growth of *Salmonella* on the day of sampling (three successive samples scheduled on 3 days in 1 week). The blue bar represents the numbers of *Salmonella* carriers detected, respectively, from stool samples on day 1 (40 of 2234; 1.8% (95% confidence interval (C.I.): 1.3–2.4), stool sample on day 2 (38 of 2224; 1.7% C.I.: 1.3–2.3), and stool sample on day 3 (41 of 2219; 1.8% C.I.: 0.9–1.8). The red bar represents the cumulative numbers of *Salmonella* carriers detected: for stool sample 2, *n* = 70 represents 40 *Salmonella* carriers detected in stool sample 1 + 30 additional *Salmonella* carriers detected in stool sample 2; for stool sample 3, *n* = 98 represents 70 *Salmonella* carriers detected in stool sample 1 and stool sample 2 + 28 additional *Salmonella* carriers detected in stool sample 3. Cumulative percentages of growth expressed for 2,219 participants were as follows: stool sample 1(40 of 2219; 1.8% C.I.: 1.3–2.4%), stool sample 2(70/2219; 3.2% C.I.: 2.5–3.9%), and stool sample 3(98/2219; 4.4% C.I.: 3.6–5.4%).

Among the 98 *Salmonella* carriers, 97 had three consecutive stool samples. The proportions of growth for each of the 3 days were similar, but cumulative carrier ratios increased from 1.8% (*n* = 40) on day 1 to 3.2% (*n* = 70) on day 2, and 4.4% (*n* = 98) on day 3, respectively (Figure 4, Supplementary Table 4). Most (82 out of 98, 83.7%) carriers had only a single sample grown with *Salmonella*, 11.2% (11 out of 98) and 5.2% (5 out of 97) had, respectively, 2 and 3 successive stool samples grown with *Salmonella*. Supplementary Document 1 summarizes the validation and user's experience of The CHROMagarTM *Salmonella* medium.

## Discussion

### Summary of findings

In an area endemic for iNTS infections, 4.4% of healthy residents were *Salmonella* carriers. Invasive iNTS (*S*. Typhimurium ST313 and Enteritidis ST11) accounted for 26 and 3 carriers, respectively, representing together 1.3% of residents living in 6.0% of households assessed. All Typhimurium and 1 out of three Enteritidis stool culture isolates had MLVA types that matched with those of blood cultures obtained in the same period from patients living in the same area. iNTS carriers were observed among all age groups. Over three-quarters of stool and blood culture isolates showed MDR, third-generation cephalosporin resistance, and fluoroquinolone non-susceptibility.

### Comparison with proportions of iNTS carriers in sub-Saharan Africa: population-based studies

Recent studies (since 2000) assessing non-typhoidal *Salmonella* carriers in sub-Saharan Africa are rare. Most of them addressed risk groups such as food handlers (Feglo et al., 2004; Smith et al., 2008; Addis et al., 2011; Misganaw and Williams, 2013; Bradbury et al., 2015), *Schistosoma*-infected individuals (Mohager et al., 2014; Salem et al., 2015; Mbuyi-Kalonji et al., 2020), or convalescent patients (Nkuo-Akenji et al., 2001), and none of them assessed sequence type or relatedness with clinical isolates.

The Typhoid Fever Surveillance in Africa Program (TSAP) found in urban populations in Guinea-Bissau and Senegal respectively 2.4% and 1.0% healthy *Salmonella* carriers, equally distributed among sex and age groups except for a higher proportion (4.2%) among the 5 – 14 years olds in Guinea-Bissau (Im et al., 2016). In contrast to the present study, no iNTS serotypes were detected, which is in line with the low incidence ratios of iNTS infections demonstrated in these countries by the TSAP study (Marks et al., 2017).

The aforementioned *Salmonella* carrier study in a *Schistosoma*-endemic area in rural DRC sampled two consecutive stool samples. It revealed that 3.4% of *Salmonella* carriers comprised 9 out of 38 (23.7%) iNTS carriers (*n* = 5 and 4 for Enteritidis and Typhimurium, respectively), most of which had MLVA types matching with those of corresponding serotypes in blood cultures obtained at Kisantu Hospital (Mbuyi-Kalonji et al., 2020). The results of the present study were similar (3.2% of *Salmonella* carriers at day 2; iNTS carriers were observed in 29.6% of carriers) but showed a higher concordance between stool and blood culture isolates (including a larger proportion of Typhimurium isolates) probably because of the neighborhood of Kisantu Hospital and by the fact that blood culture isolates were obtained from the patients living in the same health area as the study participants.

### Comparison between iNTS in stool cultures and data from other studies

Recent studies from sub-Saharan Africa provided further evidence of the presence of iNTS in stool samples, both in healthy carriers and in patients with diarrhea. In a hospital-based study in Kisantu, DRC, Phoba and coworkers found paired (same serotype and MLVA-type, closely related to WGS results) stool-blood culture iNTS isolates in 27.4% out of 299 children with iNTS bloodstream infections. Among the control group (children admitted with no febrile illness), the proportion of Salmonella carriers was comparable to that of the present study, *i.e*., 2.1% (1.6% and 0.5% for Typhimurium and Enteritidis respectively). Further, the MLVA types in the control group were identical to those from blood cultures, which was also observed in the present study.

In suburban slums in Nairobi, Kenya, Kariuki and co-workers assessed the so-called “hot spots” of iNTS bloodstream infections and found Typhimurium and Enteritidis serotypes in stool cultures from patients and healthy controls (Kariuki et al., 2019, 2020). Furthermore, in a population-based cohort study in rural Kenya, *Salmonella* Typhimurium ST313 and Enteritidis ST11 were found in stool samples of patients with diarrhea but in a lower proportion compared with blood cultures (Akullian et al., 2018).

Two studies [one in rural Burkina Faso and another in a Blantyre informal settlement (Malawi)] assessed *Salmonella* carriage in household members and livestock of index patients with *Salmonella* bloodstream infection. Both studies found matching index patient–household member pairs of *S*. Typhimurium ST313 and related ST3257, whereas livestock stool samples grew other (non-iNTS) *Salmonella* serotypes. In a study by Burkina Faso, the three Typhimurium ST313 carriers represented 1.0% out of 293 household members sampled, which is in line with the present findings (Post et al., 2019; Koolman et al., 2022).

Recent genetic analysis of *Salmonella* isolates obtained during the Global Enteric Multicenter Study (GEMS, 2007-10) confirmed widespread asymptomatic carriage of *S*. Typhimurium ST313 in children from sub-Saharan Africa (Gambia, Mali, Mozambique, and Kenya) (Kasumba et al., 2021). Finally, a retrospective analysis of *Salmonella* isolates obtained in Bangy, Central African Republic showed that in 13 patients, *S*. Typhimurium ST313 was obtained from both blood and stool isolates (Breurec et al., 2019).

### Interpretation of findings: arguments for a human reservoir of iNTS but questions remain

The present study corroborates the findings of the above cited studies pointing to a human reservoir of iNTS. To the best of our knowledge, it is the first population-based study to demonstrate the widespread occurrence of iNTS carriers, *i.e.*, 1.3% of the population, largely outnumbering the *S*. Typhi carriers. The strength of attribution of healthy humans as a reservoir for iNTS is high, given the matches between MLVA profiles and WGS clustering of stool and blood isolates. Additional evidence contributing to the human reservoir of iNTS are the similar ratios of iNTS Typhimurium versus Enteritidis among the stool and blood culture isolates and the coincidence in time between stool and blood culture isolates of the time bound MLVA types.

However, there are questions remaining about the carriage, such as the duration of shedding, factors affecting persistence (*e.g.*, hepatobiliary and urogenital tract lesions), the existence and frequency of chronic carriers, the pattern of excretion (bacterial load and intermittent excretion), and infectious dose of iNTS in vulnerable populations (Im et al., 2016; Mbuyi-Kalonji et al., 2020; Phoba et al., 2020). The few data available are data about the duration of fecal shedding obtained for diarrheagenic (non-iNTS) *Salmonella* serotypes and convalescent shedders: duration of shedding is short, with less than 2.2% of convalescent carriers excreting *Salmonella* beyond 30 days (Haeusler and Curtis, 2013; Ohad Gal-Mor, 2019). The occurrence of iNTS carriers across all ages (as presently observed) also raises the possibility of sub-clinical and unapparent iNTS infections in adults, as in the case of typhoid fever (Parry et al., 2002).

Furthermore, despite index patient–household studies and recent food chain studies in Tanzania and Kenya failed to demonstrate iNTS isolates in animal and environmental samples (Kariukiet al., 2006; Dione et al., 2011; Post et al., 2019; Wilson et al., 2020; Crump et al., 2021; Koolman et al., 2022), most authors concluded that an environmental reservoir for iNTS is not yet excluded (Mather et al., 2015; Post et al., 2019; Crump et al., 2021; Kasumba et al., 2021; Koolman et al., 2022). A recent study from Kisangani (DRC) showed that urban rats harbored ST313 isolates which genetically matched with blood culture isolates (Falay et al., 2022), and another study in Kisantu found a direct and short-cycle type association between rainfall and iNTS (independent from *P. falciparum* malaria), suggesting flooding of water and food by contaminated surface water as an option of transmission (Tack et al., 2021). Other indirect evidence of environmental risk factors—observed in Kenya and Burkina Faso—included the vicinity of unimproved water sources, water vending points, and sewage systems, as well as consumption of street food (Kariuki et al., 2019; Mbae et al., 2020; Nikiema et al., 2021).

### Relevance and generalizability of findings

The evidence of a human reservoir and the relatively high (1.3%) proportion of iNTS carriers in the community may orient and prioritize control measures such as vaccines (Im et al., 2016; Kariuki et al., 2019). The present study was conducted in a setting with high NTS but low HIV burden and in an area with a moderate population density but close to a major national road. DRC is one of the few countries in sub-Saharan Africa that did not experience a decline in the *P. falciparum* malaria burden (World Health Organization, 2022). Therefore, the present findings are probably not generalizable to other settings and carriers; reservoir and transmission studies should be conducted in settings with different iNTS burdens and associated risk factors.

### Antimicrobial resistance profile

Invasive iNTS are known for their association with antimicrobial resistance (Tack et al., 2020b). In the present study, the most worrying was the combination of MDR, resistance to third-generation cephalosporins, and FQNS in *S*. Typhimurium, which can be categorized as extensive drug resistance and leaves few therapeutic options (Tack et al., 2020b). It contrasts with the pan-susceptible *S*. Typhimurium lineage 3 which emerged in Malawi in 2016 (Pulford et al., 2021). Very recently, the World Health Organization listed efficacious and safe antibiotic treatment regimens for drug-resistant *Salmonella* among the 40 priorities on the Global Research Agenda for Antimicrobial Resistance in Human Health, thereby explicitly referring to fluoroquinolone and third-generation cephalosporin resistance (World Health Organization, 2023).

The very low to absent resistance among most of the non-iNTS *Salmonella* serotypes (excluding *S*. Kentucky, see below) has also been observed in the aforementioned rat carrier study from Kisangani (Falay et al., 2022). These observations add to the probability of the human-confined habitat of ST 313 Typhimurium.

### Other findings (secondary objectives)

Culturing three consecutive stool samples provided a 2.5 times higher yield than culturing a single sample (4.4 vs. 1.8%, respectively). This practice of three consecutive stool samples is based on a small-scale study detecting chronic carriers of typhoid fever (Anderson et al., 1961) and, to our knowledge, has not been validated before. In the present study, if only the first stool sample per participant was cultured, 59.2% (58 out of 98) of *Salmonella* carriers including 76.9% (20 out of 26) of *S*. Typhimurium would have been missed (Supplementary Table 4). As to laboratory work-up, the CHROMagar^TM^ *Salmonella* proved to be reliable and user-friendly.

In line with other anecdotal observations (Im et al., 2016; Post et al., 2019; Koolman et al., 2022), *Salmonella* isolates (including *S*. Typhimurium) tended to cluster in households. These observations highlight the transmissibility of non-typhoidal *Salmonella* including iNTS and may provide support to the “hot spot” concept of iNTS transmission as a priority for the implementation of iNTS vaccines (Kariuki et al., 2019). In the present study, the spectrum of *Salmonella* serotypes other than iNTS differed from the earlier *Schistosoma-Salmonella* study in the same province in DRC (Mbuyi-Kalonji et al., 2020) and may reflect local or over-time evolutions. The preponderance of *Salmonella* Kentucky is striking; MDR *S*. Kentucky ST198 has spread worldwide through human traveling and poultry trade (Hello et al., 2013; Dieye et al., 2022) but was previously not reported from DRC. The ciprofloxacin MIC values of *S*. Kentucky were strikingly high, suggesting the accumulation of resistance genes and a food chain-related reservoir.

### Limitations and strengths

Limitations inherent to the study design are the “snapshot” design, which precludes interpretation of transmission. The study was conducted during the rainy season, which is associated with an increase in iNTS infections (Tack et al., 2021). However, in the aforementioned hospital-based iNTS carrier study in Kisantu, the proportions of stool cultures grown with iNTS were similar across the rainy and dry seasons (Phoba et al., 2020). Furthermore, there were risks of bias by the non-availability of working-age adult men, but, in anticipation of this risk, household visits were conducted early morning, and the included participants had similar demographic profiles as the total population of Kikonka. Finally, to assess household clustering of *Salmonella* carriers, the sample selection was based on households and not on individuals. Household clustering may have increased the number of detected carriers, but, in turn, this risk may be mitigated by the high number of households enrolled.

Strengths of the present study included the recent census (guiding selection of households), the large sample size, and the representation of participants for the total Kikonka population and geography of the area. In addition, the participation ratio was as high as 94.9%, compared to 82.9% and 72.7% previously reported in Guinea-Bissau and Senegal (Im et al., 2016). Further, the compliance with 3-day sampling was excellent and much higher compared to 85.0% for a 2-day sampling previously (Mbuyi-Kalonji et al., 2020). Furthermore, different colony types were assessed (allowing to detect mixed infections) and quality indicators were monitored. The high consistency of proportions across visit rounds and days of sampling illustrated the robustness of procedures (Koolman et al., 2022).

## Conclusion

In conclusion, the present study demonstrated iNTS (Typhimurium ST313 and in a lower proportion Enteritidis ST11) carriage among healthy community members in an iNTS endemic area. The stool iNTS isolates were genetically similar to blood culture isolates from patients obtained in the same place and time frame. The present findings complement the growing evidence of humans as the reservoir of iNTS, but further studies are needed to understand transmission and explore co-existent animal or environmental reservoirs.

## Data availability statement

The data presented in the study are deposited in the European Nucleotide Archive (ENA) repository, accession number PRJEB64271.

## Ethics statement

The studies involving humans were approved by The Institutional Review Board of the Institute of Tropical Medicine (Reference 1263/18). The Ethical Committee of the University of Antwerp (Reference 18/47/535). The Ethical Committee of Public Health School in Kinshasa, Democratic Republic of the Congo (ESP/EC/070/2019). Ethical approval for the blood culture surveillance study was granted by the Institutional Review Board of the Institute of Tropical Medicine (Reference 08 17 12 613) and the Ethics Committee of Public Health school in Kinshasa (version 1: ESP/CE/073/2015 and version 2: ESP/CE/074/2015). For the SETA study, ethical approvals were granted by the International Vaccine Institute Institutional Review Board (IRB No. 2015-006) and the Ethics Committee of Public Health school in Kinshasa (version 1.1: ESP/CE/011/2017, version 1.2: ESP/CE/011B/2017 and version 1.3: ESP/CE/037/2018 and ESP/CE/037B/2019). The studies were conducted in accordance with the local legislation and institutional requirements. Written informed consent for participation in this study was provided by the participants' legal guardians/next of kin. Written informed consent was obtained from the individual(s), and minor(s)' legal guardian/next of kin, for the publication of any potentially identifiable images or data included in this article.

## Author contributions

LM-K: Conceptualization, Data curation, Formal analysis, Investigation, Methodology, Software, Visualization, Writing—original draft, Writing—review & editing. LH: Data curation, Formal analysis, Investigation, Methodology, Resources, Validation, Writing—original draft, Writing—review & editing. JM: Investigation, Resources, Writing—review & editing. M-FP: Conceptualization, Investigation, Methodology, Project administration, Resources, Supervision, Validation, Writing—review & editing. GN: Investigation, Resources, Writing—review & editing. WM: Data curation, Formal analysis, Investigation, Methodology, Resources, Writing—original draft, Writing—review & editing. JI: Funding acquisition, Project administration, Resources, Writing—review & editing. FM: Funding acquisition, Project administration, Resources, Supervision, Writing—review & editing. HJ: Funding acquisition, Resources, Writing—review & editing. JJ: Conceptualization, Formal analysis, Funding acquisition, Methodology, Project administration, Resources, Software, Supervision, Validation, Visualization, Writing—original draft, Writing—review & editing. OL: Conceptualization, Funding acquisition, Methodology, Project administration, Resources, Supervision, Validation, Writing—review & editing.

## Footnotes

^1^ https://enterobase.warwick.ac.uk/
