# Supplementary material for: Invasive non-typhoidal Salmonella from stool samples of healthy human carriers are genetically similar to blood culture isolates: a report from the Democratic Republic of the Congo
Source: Front Microbiol. 2023 Nov 24;14:1282894. doi: 10.3389/fmicb.2023.1282894 (PMC10704266; doi:10.3389/fmicb.2023.1282894)
Supplement: Supplementary file 1 [file Table_1.DOCX]

**Supplementary Table 1.** **Age and gender distribution of the total population of Kikonka health area as determined by the census.**

| Age groups | F | M | Total |  | Proportion of the total population of Kikonka health area* |
| --- | --- | --- | --- | --- | --- |
| <2 years | 466 | 457 | 923 | Children < 5 years | 16.3% |
| 2 -< 5 years | 888 | 878 | 1,766 |  |  |
| 5 -< 10 years | 1,307 | 1,377 | 2,684 | Children < 15 years | 30.5% |
| 10 -< 15 years | 1,097 | 1,245 | 2,342 |  |  |
| 15 -< 20 years | 921 | 829 | 1,750 | Adults ≥ 15 years | 53.2% |
| 20 -< 30 years | 1,119 | 937 | 2,056 |  |  |
| 30 -< 40 years | 970 | 787 | 1,757 |  |  |
| 40 -< 50 years | 702 | 702 | 1,404 |  |  |
| ≥ 50 years | 979 | 842 | 1,821 |  |  |
| Total | **8,449** | **8,054** | **16,503** |  | **100%** |

*Percentages calculated with the total population in the column

Median age (IQR): 16 years (7 – 34)

M/F ratio was 1:1.05

16,503 residents living in 3,218 households

Median number of household members per household (range) was 6 (1 – 19).
