# Supplementary material for: Invasive non-typhoidal Salmonella from stool samples of healthy human carriers are genetically similar to blood culture isolates: a report from the Democratic Republic of the Congo
Source: Front Microbiol. 2023 Nov 24;14:1282894. doi: 10.3389/fmicb.2023.1282894 (PMC10704266; doi:10.3389/fmicb.2023.1282894)
Supplement: Supplementary file 2 [file Table_2.DOCX]

**Supplementary Table 2. Age and gender distribution of the participants (= eligible subjects who consented and provided at least one stool sample).**

| **Age groups** | **F** | **M** | **Total** |  | **Proportions of participants*** |
| --- | --- | --- | --- | --- | --- |
| **< 2 years** | 55 | 57 | 112 | Children < 5 years | 15.9% |
| **2 -< 5 years** | 123 | 120 | 243 |  |  |
| **5 -< 10 years** | 195 | 193 | 388 | Children < 15 years | 30.3% |
| **10 -< 15 years** | 135 | 155 | 290 |  |  |
| **15 -< 20 years** | 113 | 119 | 232 | Adults ≥ 15 years | 53.8% |
| **20 -< 30 years** | 151 | 115 | 266 |  |  |
| **30 -< 40 years** | 126 | 81 | 207 |  |  |
| **40 -< 50 years** | 106 | 97 | 203 |  |  |
| **≥ 50 years** | 158 | 135 | 293 |  |  |
| **Total** | **1,162** | **1,072** | **2,234** |  | **100%** |

*Percentages calculated with the total population in the column

Median age (IQR): 16 years (7 – 37)

M/F ratio was 1:1.08

2,234 subjects living in 482 households

Median number of household members (range) per household was 5 (1 – 12).
