# Supplementary material for: Invasive non-typhoidal Salmonella from stool samples of healthy human carriers are genetically similar to blood culture isolates: a report from the Democratic Republic of the Congo
Source: Front Microbiol. 2023 Nov 24;14:1282894. doi: 10.3389/fmicb.2023.1282894 (PMC10704266; doi:10.3389/fmicb.2023.1282894)
Supplement: Supplementary file 3 [file Table_3.DOCX]

**Supplementary Table 3. Age distribution of subjects not solicited and reason why they were not solicited**

| **Age groups** | **Absent from the site** | **Died** | **Total** |  | **Proportions of total eligible subjects *** |
| --- | --- | --- | --- | --- | --- |
| **< 2 years** | 31 | 2 | 33 | Children < 5 years | 15.0% |
| **2 -< 5 years** | 42 | 2 | 44 |  |  |
| **5 -< 10 years** | 69 | 0 | 69 | Children < 15 years | 25.2% |
| **10 -< 15 years** | 59 | 1 | 60 |  |  |
| **15 -< 20 years** | 70 | 0 | 70 | Adults ≥ 15 years | 59.8% |
| **20 -< 30 years** | 102 | 0 | 102 |  |  |
| **30 -< 40 years** | 54 | 0 | 54 |  |  |
| **40 -< 50 years** | 24 | 2 | 26 |  |  |
| **≥ 50 years** | 52 | 3 | 55 |  |  |
| **Total** | **503** | **10** | **513** |  | **100%** |

*Percentages calculated with the total population in the column

Median age (IQR): 18 years (8 – 31)

M/F ratio was 1:0.82

513 subjects living in 226 households

Median number of absents per household (range) was 1 (1 – 8) household members.
