# Supplementary material for: Invasive non-typhoidal Salmonella from stool samples of healthy human carriers are genetically similar to blood culture isolates: a report from the Democratic Republic of the Congo
Source: Front Microbiol. 2023 Nov 24;14:1282894. doi: 10.3389/fmicb.2023.1282894 (PMC10704266; doi:10.3389/fmicb.2023.1282894)
Supplement: Supplementary file 4 [file Table_4.DOCX]

**Supplementary Table 4. *Salmonella* serotypes among carriers detected by stool cultures on day 1, day 2 and day 3 (data for 98 carriers and 101 serotypes).** Numbers of additional carriers represent carriers of serotypes that had not been detected the previous day(s).

| Serotypes | Number of carriers on day 1 (Number of households) | Number of additional carriers on day 2  (Number of households) | Number of additional carriers on day 3  (Number of households) |
| --- | --- | --- | --- |
| *Salmonella* Kentucky | 23 (14) | 11 (4) | 15 (6) |
| *Salmonella* Typhimurium | 6 (6) | 12 (4) | 7***** (7) |
| *Salmonella* II 42:r:- | 7 (5) | 3 (3) | 5****** (3) |
| *Salmonella* Urbana | 2 (2) | 2 (1) | 0 (0) |
| *Salmonella* Enteritidis | 0 | 1 (1) | 2 (2) |
| *Salmonella* Tempe | 1 (1) | 0 | 0 |
| *Salmonella* Typhi | 1 (1) | 0 (0) | 0 (0) |
| *Salmonella* I 11:-:1,2 | 0 | 1 (1) | 0 |
| Total | **40 (29)** | **30 (14)** | **28** (18)** |

*****Two *Salmonella* carriers had *S.* Typhimurium and *S.* Urbana isolated from different stool samples

******One *Salmonella* carrier had *S.* Typhimurium and *Salmonella* II 42:r:- in the same stool sample.
