# Supplementary material for: Invasive non-typhoidal Salmonella from stool samples of healthy human carriers are genetically similar to blood culture isolates: a report from the Democratic Republic of the Congo
Source: Front Microbiol. 2023 Nov 24;14:1282894. doi: 10.3389/fmicb.2023.1282894 (PMC10704266; doi:10.3389/fmicb.2023.1282894)
Supplement: Supplementary file 5 [file Table_5.DOCX]

**Supplementary document 1. Validation and field performance of the CHROMagar^TM^ *Salmonella* medium.**

CHROMagar^TM^ *Salmonella*: validation, production and quality control

Prior to field use, the CHROMagar^TM^ *Salmonella* medium (CHROMagarTM, Paris, France Reference: SA132, lot: PO01131) was validated at the National Institute of Biomedical Research (INRB, Kinshasa, Democratic Republic of the Congo) by inoculating 22 bacterial species comprising 14 Enterobacterales (including 6 *Salmonella* enterica serotypes: two *Salmonella* enterica serotype Typhi, one *Salmonella* Typhimurium, one *Salmonella* Enteritidis and two *Salmonella* sp.), 5 *Staphylococcus* *aureus* strains and 3 non-fermenting bacteria both as pure cultures and as contrived stool samples. When inoculated on CHROMagar^TM^ *Salmonella* medium, all 6 *Salmonella* isolates yielded purple colonies when read at 24 hours of incubation at 35°C. Further, after 48 hours of incubation, the *Salmonella* colonies remained purple while other Enterobacterales yielded blue colonies. After 24 hours of incubation, *Acinetobacter* displayed white colonies which turned to purple after 48 hours of incubation. *Pseudomonas aeruginosa* and *Staphylococcus aureus* did not grow.

During the field study at Kisantu hospital, CHROMagar^TM^ plates were prepared at Kisantu Hospital according to the manufacturer's instructions; quality control was done with *Salmonella* Typhimurium ATCC 13311 and *Escherichia coli* ATCC 25922. Poured plates were stored between 2 – 8°C and protected from light. All stool samples that grew with *Salmonella* displayed purple colonies on CHROMagar^TM^ after 24 hours of incubation; these colonies remained purple at 48 hours of incubation. Prolonged (48 hours) incubation of samples that had no sign of growth at 24 hours did not reveal additional *Salmonella* isolates. At 24 hours of incubation, 97/2,234 (4.3%) samples displayed purple colonies which were not confirmed as *Salmonella*; these colonies had notably smaller sizes than the *Salmonella* isolates which made them easier to distinguish over time.
